# Biomechanical analysis of vertebral wedge deformity in elderly women with quantitative CT-based finite element analysis

**DOI:** 10.1186/s12891-022-05518-z

**Published:** 2022-06-14

**Authors:** Jing Liu, Xiaodong Cheng, Yan Wang, Ping Zhang, Lei Gao, Xingyuan Yang, Shaoqiang He, Ying Liu, Wei Zhang

**Affiliations:** 1 Department of Radiology, The Third Hospital of Hebei Medical University, No. 139 Ziqiang St, Qiaoxi District, Shijiazhuang, Hebei CN 050050 China; 2 Key Laboratory of Biomechanics of Hebei Province and Orthopaedic Research Institution of Hebei Province, Shijiazhuang, 050000 Hebei China; 3 Department of Endocrinology, The Third Hospital of Hebei Medical University, No. 139 Ziqiang St, Qiaoxi District, Shijiazhaung, Hebei CN 050000 China; 4 Department of CT/MRI, The Third Hospital of Hebei Medical University, No. 139 Ziqiang St, Qiaoxi District, Shijiazhuang, Hebei CN 050050 China

**Keywords:** Biomechanical analysis, Vertebral wedge deformity, Quantitative CT, Finite element analysis

## Abstract

**Background:**

To explore the vertebral deformity angle (VD angle) of 1^st^ lumbar vertebral body (L1) in elderly women, investigate the influence of VD on vertebral stiffness (VS) by biomechanical analysis using quantitative computed tomography-based finite element analysis (QCT-FEA).

**Methods:**

Two hundred seventy eight participants were recruited, and underwent QCT scan. Measured VD angles of L1, and constructed QCT-FEA models of L1 with the minimum (0.59°), median (5.79°) and maximum (11.15°) VD angles, respectively. Loads in two directions were applied on the upper edge of L1 with a force of 700 N, and vertebral stiffness (VS) was defined as the ratio of 700 N and displacement at the superior reference point: (1) perpendicular to the upper edge of L1 (defined as VS-U); (2) perpendicular to the lower edge of L1(defined as VS-L).

**Results:**

Age was very weak positively correlated with VD angle, moderate negatively correlated with vBMD, and moderate negatively correlated with VS (*P* < 0.05). VS-U was significantly different among three VD angles, so was VS-L (*P* < 0.001). VS-U was higher than VS-L in 5.79° and 11.15° VD angles (*P* < 0.05), however no difference in 0.59° VD angles (*P* > 0.10).

**Conclusions:**

VD angle of L1 was slightly increased with age and not correlated with vBMD, and VS was moderate negatively correlated with age, showing that the vertebral body was more likely to fracture with aging. VS-U and VS-L were gradually decreased with the increase of VD angle, and VS-L was lower than VS-U with the increase of VD angle, which showed that vertebral body was more prone to fracture when the load was perpendicular to the lower edge of the vertebral body as the VD angle increasing.

**Supplementary information:**

The online version contains supplementary material available at 10.1186/s12891-022-05518-z.

## Background

Vertebral deformity (VD) is mainly attributable to osteoporosis and classical hallmark of osteoporosis, associated with marked increase in morbidity, mortality and health economic burden [1]. There are three types of VD, crush, wedge and biconcave, of which the most common type is controversial [2–4]. Some studies showed that wedge deformity is the most frequent and tends to cluster at the mid-thoracic and thoracolumbar regions of the spine [2, 5–7], while some studies suggested that biconcavity is the most common type [8, 9]. VD is increased with age and more marked in women [2], because there is often no remembered injury and visible fracture plane on radiographs [1, 10], and the term “deformity” is often used rather than “fracture” [11]. Previous studies suggest that gradual time-dependent “creep” processes may contribute to VD, which is continuing deformation under constant load [10, 12, 13]. Some studies regarding restoration of vertebral height had been reported [14–16]. However, few studies focus on the influence of VD on vertebral stiffness (VS).

Quantitative computed tomography (QCT) has become the most common tool for measuring volume bone mineral density (vBMD), which can avoid the influence of vertebral osteophyte, facet degeneration, intervertebral disc stenosis, endplate sclerosis and abdominal aortic wall calcification [17–19]. However, given the fact that fracture is a biomechanical phenomenon by its nature, bone mineral density (BMD) may not be the most effective tool for fracture risk assessment [20]. QCT-based finite element analysis (QCT-FEA) promises an improved tool for assessing fracture-related mechanical characteristics (e.g.: stiffness and strength) on patient-specific basis accounting for BMD [21–24].

The purpose of our study was to explore the vertebral deformity angle (VD angle) of 1^st^ lumbar vertebral body (L1) in elderly women, and investigate the influence of VD angle on VS by biomechanical analysis using QCT-FEA.

## Methods

### Patient population

We retrospectively reviewed a single-center database of QCT examination from January 2017 to December 2019 with approval from the local institutional review board (2018–036-1). Written informed consent was provided to all participants before QCT examination. Inclusion criteria: women from 50 to 80 years old and underwent MRI examination within one week interval from QCT to exclude L1 vertebral compression fracture. Exclusion criteria: vertebral fracture and/or surgery, and biconcave or crush deformity of L1. Age, height and weight were recorded, and height and weight were measured by standard method. Body mass index (BMI) was defined as weight (kg) divided by height squared (m^2^).

### Image acquisition

All participants underwent cross-sectional CT scan of L1 (from the 12^th^ thoracic to 2^nd^ lumbar vertebra) in the supine position by using CT scanner (Somatom Sensation 64, Siemens, Erlangen, Germany) with hands above the head and a solid Mindways QCT phantom (Mindways Software Inc., Austin, TX, USA) closely dorsal side of each participant simultaneously. CT scan was performed using the following parameters: 120 kV, 125 mAs, 168 cm table height, 512 × 512 matrix, 1 mm slice thickness, and 500 mm field of view.

### Image analysis

#### VD angle

**Fig. 1:**
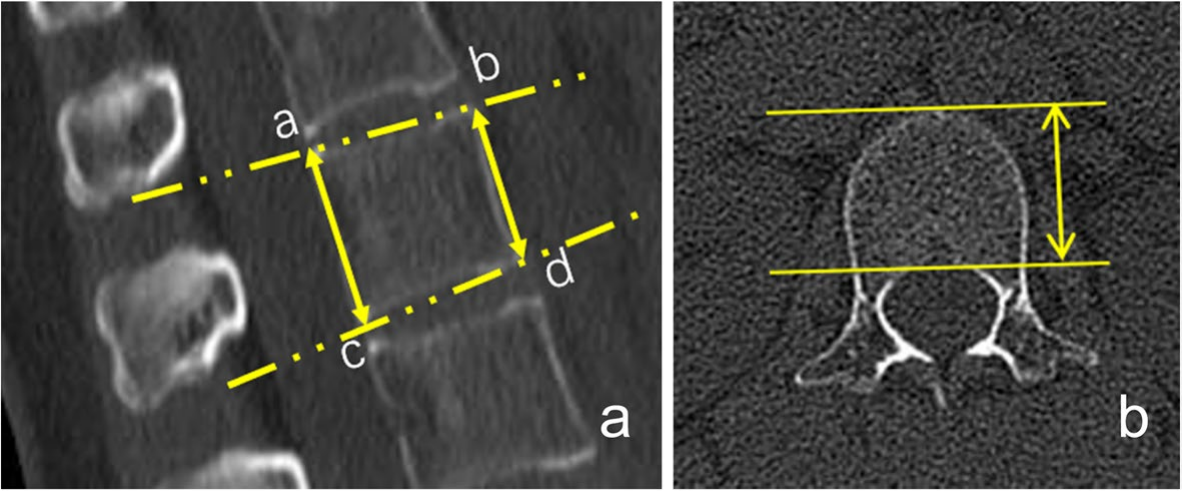
Schematic diagram of VD angle measurement of L1. **a**: Four marginal points (a, b, c and d) of the maximum sagittal plane of the L1 vertebral body were established, and distance between point a and point **c** (identified D_ac_), and distance between point **b** and point **d** (identified D_bd_) were measured. **b**: The tangent line of the anterior arc at the maximum plane of the axial L1 vertebral body was identified as the anterior edge line, and the parallel line of the anterior edge line and across the posterior margin of the vertebral body was identified as the posterior edge line. Distance between the anterior edge line and the posterior edge line was identified D_ap_. VD angle = (D_ac_—D_bd_)/ D_ap_. VD angle: vertebral deformity angle. L1: 1^st^ lumbar vertebral body

**Fig. 2:**
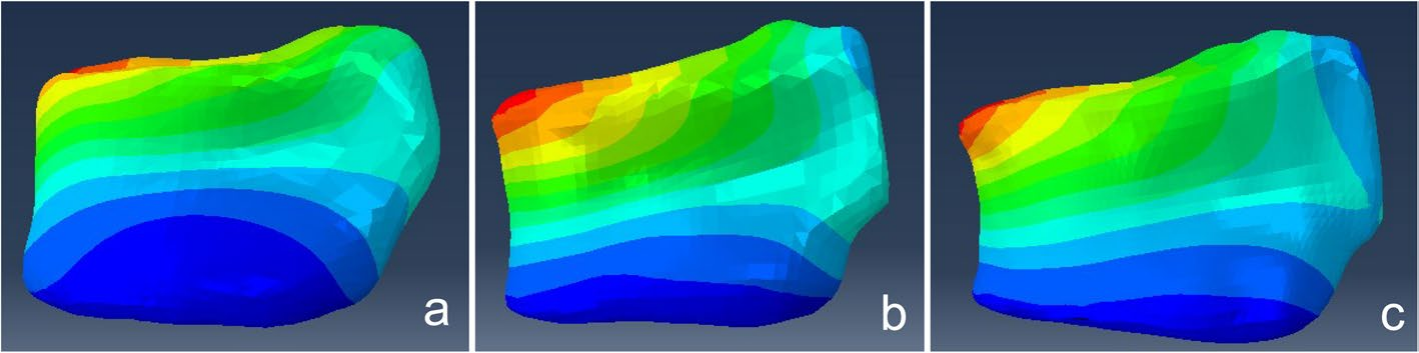
QCT-FEA models with three different VD angles of L1. **a**: QCT-FEA model with VD angle of 0.59 degrees. **b**: QCT-FEA model with VD angle of 5.79 degrees. **c**: QCT-FEA model with VD angle of 11.15 degrees. QCT-FEA: quantitative computed tomography-based finite element analysis. VD angle: vertebral deformity angle. L1: 1^st^ lumbar vertebral body

The measurement method of VD angle was shown in Fig. 1, and VD angles were measured by two analysts (a primary analyst, Y.L., with 5 years of imaging diagnostic experience, and a secondary analyst, J. L., with 3 years of imaging diagnostic experience), and then took their average for further used. VD angles of 278 participants were not normal distribution, and we constructed QCT-FEA models of L1 with the minimum (0.59°), median (5.79°) and maximum (11.15°) VD angles, respectively (Fig. 2).

#### vBMD

Images were transferred to a QCT workstation and analyzed using the three-dimensional spine function version 5.10 of Mindways QCT pro soft-ware (Mindways Software Inc., Austin, TX, USA). Regions of interest (ROI) about 250 mm^2^ area and 9 mm height was placed at the midplane of L1 vertebral body to avoid the influence of cortical bone and proliferative osteophyte. vBMD were measured by two radiologists, and then took the average for analysis.

#### QCT-FEA models

We constructed QCT-FEA models of L1 using the Mimics program (Mimics 21.0, Materialize Inc, Leuven, Belgium) and Geomagic Studio 2013 (Geomagic, Inc., Research Triangle Park, NC, USA). Three-dimensional finite element models were built by converting each voxel in the QCT images directly into a linear cube-shaped tetrahedral element. QCT images of DICOM format were imported into Mimics, vertebral bone was segmented out, and then coronal, sagittal and axial images of lumbar vertebra were obtained. Threshold, split mask, edit masks, calculate three dimensions and other steps were performed of L1 to fill the holes and remove the sharp angles, and then a preliminary three-dimensional geometric model was obtained. The preliminary three-dimensional geometric model was imported into Geomagic Studio of STL format, smoothened, and then exported into STP/STEP format. The spine structure is complex, so in this study, the vertebral body accessory, intervertebral disc, ligament muscle and other structures were removed, and only the vertebral body was analyzed for the need to simplify the models. In Abaqus software, material properties of L1 were endowed. The material property (e.g., elastic modulus) of each element was automatically mapped from the corresponding voxel of the CT dataset based on its Hounsfield unit (HU) [25, 26]. The relationship between HU and QCT-measured BMD (ρQCT, mg/cm^3^) has been assumed linear [27, 28], and was determined based on the calibration phantoms:

$$\rho \mathrm{QCT }=0.5325\mathrm{Hu}-38.401$$

An empirical relationship between BMD and elastic modulus (E, MPa), which was previously established based on mechanical testing of the human vertebral cadavers, was used to determine the material properties of the bone elements [29, 30]:

$$E={4730\rho QCT}^{1.56}$$

The Poisson’s ratio was set to 0.3, as commonly used in bone QCT-based finite element models [22, 31].

#### Biomechanical analysis

**Fig. 3:**
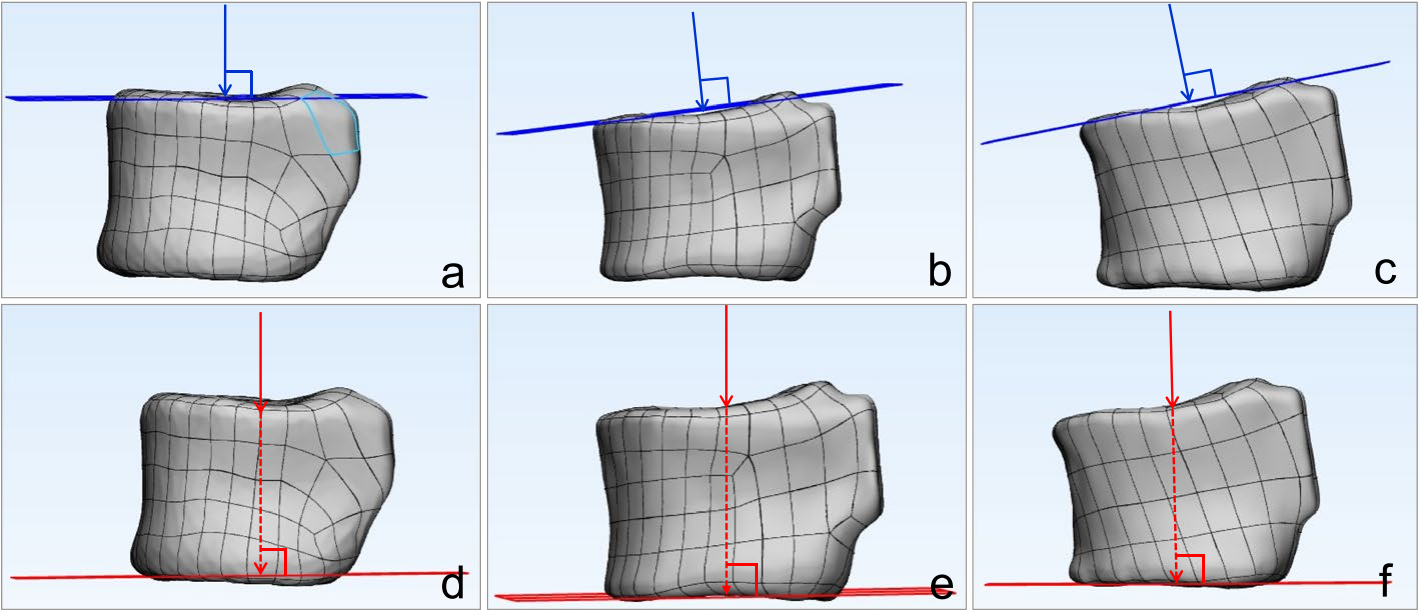
Schematic diagram of different directional loads applied on the QCT-FEA models. **a** and **d**: QCT-FEA model with VD angle of 0.59 degrees. **b** and **e**: QCT-FEA model with VD angle of 5.79 degrees. **c** and **f**: QCT-FEA model with VD angle of 11.15 degrees. **a**, **b** and **c**: Loads perpendicular to the upper edge of L1 were applied to the upper edge of L1 with a force of 700 N. **d**, **e** and **f**: Loads perpendicular to the lower edge of L1 were applied to the upper edge of L1 with a force of 700 N. QCT-FEA: quantitative computed tomography-based finite element analysis. VD angle: vertebral deformity angle. L1: 1^st^ lumbar vertebral body

Abaqus software (Abaqus 6.13, Dassault Systèmes, RI, USA) was used for biomechanical analysis of three angles QCT-based finite element model among each participant, respectively. The boundary was set, and freedom of all nodes on the lower surface of L1 was set to 0. Loads in two directions were applied on the upper edge of L1 with a force of 700 N centrally:(1) perpendicular to the upper edge of L1; (2) perpendicular to the lower edge of L1 (Fig. 3).

Previous study showed that the force of 700 N represented approximately the upper limit of the linear portion of the load–displacement curve derived from all vertebral specimens in vitro cadaver experiment, and VS was defined as the ratio of the applied force (700 N) and its resulted displacement at the superior reference point [22]. VS when the load perpendicular to the upper edge of L1 was defined as VS-U, and the load perpendicular to the lower edge of L1 was defined as VS-L.

### Statistical analysis

Statistical analyses were performed using SPSS version 26.0. Shapiro–Wilk (SW) test was used to test whether the data accorded with normal distribution, and *P* < 0.05 was considered significant. Statistical description of the quantitative variables was expressed as mean ± standard deviation (SD) or median (P_25_, P_75_). Pearson or spearman correlation test was used to test the correlation among age, BMI, VD angle, vBMD and VS, and *P* < 0.05 was considered significant. Kruskal–Wallis H test was used to test the difference of VS-U among three VD angles (*P* < 0.05 was considered significant), and then Mann–Whitney U test for pairwise comparison, and statistical test criteria for pairwise comparison was calibrated as α’ = α/3, that was 0.05/3. Statistical analysis method of VS-L is the same as that of VU-U. Two independent Mann–Whitney U test was used to compare the difference between VS-U and VS-L of the same VD angle, and *P* < 0.05 was considered significant.

## Results

### Descriptive statistics and correlation analysis

Two hundred seventy eight female participants were included (Table 1).

**Table 1:** Descriptive characteristics of the female participants

	All participants
*n*	278
Age (years)	63.8 ± 8.4
BMI (Kg/m^2^)	25.3 ± 3.7
vBMD(mg/cm^3^)	94.1(68.5, 114.9)
VD angle	5.74 ± 1.91
QCT-FEA model with VD angle of 0.59 degrees	
VS-U	3838.8(2337.8, 5240.5)
VS-L	3864.2(2353.3, 5273.1)
QCT-FEA model with VD angle of 5.79 degrees	
VS-U	3198.5(1948.0, 4365.5)
VS-L	2378.9(1436.9, 3246.4)
QCT-FEA model with VD angle of 11.15 degrees	
VS-U	3038.2(1850.1, 4145.7)
VS-L	1892.7(1152.7, 2582.8)

Note: *BMI* Body mass index, *vBMD* Volume bone mineral density, *VD* angle Vertebral deformity angle of L1, *QCT-FEA* Quantitative computed tomography-based finite element analysis, *VS-U* Vertebral stiffness when the load perpendicular to the upper edge of 1^st^ lumbar vertebral body, *VS-L* Vertebral stiffness when the load perpendicular to the lower edge of 1^st^ lumbar vertebral body

Age was not correlated with BMI (*r* = -0.012, *P* = 0.842), very weak positively correlated with VD angle (*r* = 0.191, *P* = 0.001), moderate negatively correlated with vBMD (*r*_*s*_ = -0.481, *P* < 0.001), and moderate negatively correlated with VS (*r*_*s*_ raged from -0.479 to -0.481, *P* all < 0.001).

BMI was not correlated with VD angle (*r* = -0.018, *P* = 0.762), not correlated with vBMD (*r*_*s*_ = 0.093, *P* = 0.121), and not correlated with VS (*r*_*s*_ raged from 0.092 to 0.097, *P* all > 0.05).

VD angle was not correlated with vBMD (*r*_*s*_ = -0.017, *P* = 0.778), and not correlated with VS (*r*_*s*_ raged from -0.017 to -0.018, *P* all > 0.05).

### Comparison of VS

**Fig. 4:**
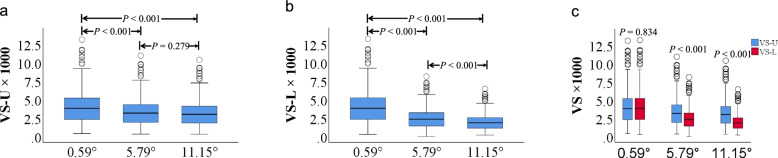
Box plots for the VS comparison. **a**: VS-U distribution among three VD angles. Kruskal–Wallis H test was used to test the difference, and then Mann–Whitney U test for pairwise comparison, and statistical test criteria for pairwise comparison was calibrated as α’ = α/3, that was 0.05/3. **b**: VS-L distribution among three VD angles. Kruskal–Wallis H test was used to test the difference, and then Mann–Whitney U test for pairwise comparison, and statistical test criteria for pairwise comparison was calibrated as α’ = α/3, that was 0.05/3. **c**: Two independent Mann–Whitney U test was used to test the difference between VS-U and VS-L of the same VD angle and statistical test criteria was α, that was 0.05. VS: vertebral stiffness. VS-U: VS when the load perpendicular to the upper edge of L1. VD angle: vertebral deformity angle. L1: 1^st^ lumbar vertebral body. QCT-FEA: quantitative computed tomography-based finite element analysis. VS-L: VS when the load perpendicular to the lower edge of L1

VS-U was significantly different among three VD angles (*Z* = 556.000, *P* < 0.001), and then for pairwise comparison, there were difference between 0.59° and 5.79°, between 0.59° and 11.15°, and no difference between 5.79° and 11.15° VD angles (Fig. 4a).

VS-L was significantly different among three VD angles (*Z* = 548.029, *P* < 0.001), and then for pairwise comparison, there were difference between 0.59° and 5.79°, between 0.59° and 11.15°, and between 5.79° and 11.15° VD angles (Fig. 4b).

VS-U was higher than VS-L in 5.79° and 11.15° VD angles (*P* < 0.05), however no difference in 0.59° VD angles (*P* > 0.10, Fig. 4c).

## Discussion

Present study showed that age was very weak positively correlated with VD angle, consistent with previous study, which showed that VD was increased with age and was more marked in women [2]. Age was moderate negatively correlated with vBMD, and the loss of BMD in women consists of two stages, slow constant age-related loss and quick oestrogen-dependent process, which begins after the menopause. Hormonal imbalance, ageing, environmental factors, life style, and genetic predispositions are responsible for about 50–80% of BMD loss [32]. Previous studies showed that typical anterior wedge deformity can be created consistently in cadaveric vertebrae in a 2-stage process. First, compressive overload fractures the endplate, decompresses the adjacent intervertebral disc, and concentrates loading onto the anterior cortex. Second, cyclic loading in flexion causes progressive collapse of the anterior cortex [11]. In our study, VD angle was not correlated with vBMD. This may be pseudomorph that increased vBMD caused by trabecular insertion of microfracture after vertebral wedge deformity, or the fact that vertebral wedge deformity mainly involves bone density under the endplate of the vertebral body, while QCT measures the vBMD of cancellous bone in the center of the vertebral body, without zonal measurement of BMD. Previous study showed that trabecular density is lower in the anterior versus posterior regions of the vertebral centrum [33], micro trabecular fractures and endplate fractures are commonly seen in osteoporotic vertebral bodies, and often in vertebrae that appeared to be uninvolved on specimen radiographs [34].

**Fig. 5:**
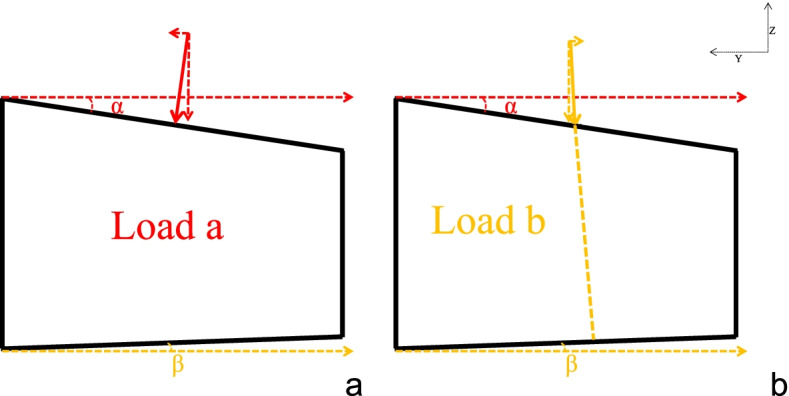
Schematic diagram of L1 vertebral body anterior wedge deformity of sagittal plane. Y axis and Z axis are the human coordinate system. **a**: Load a (solid red line with arrow) perpendicular to the upper edge of L1 was applied to the upper edge of L1 with a force of 700 N. **b**: Load b (solid yellow line with arrow) perpendicular to the lower edge of L1 was applied to the upper edge of L1 with a force of 700 N. The angle between the upper edge of L1 and Y axis was defined as angle α. The angle between the lower edge of L1 and Y axis was defined as angle β. L1: 1^st^ lumbar vertebral body

The density-modulus relationship was directly adopted from a previous study where mechanical testing was conducted on vertebral bone of white subjects; thus, the predictive abilities of our QCT-FEA models for stiffness are well validated [22, 29, 30]. VS was defined as the ratio of the applied force (700 N) and its resulted displacement at the superior reference point, reflecting the ability of vertebral body to resist fracture to some extent. In present study, VS was moderate negatively correlated with age, showing that the vertebral body was more likely to fracture with aging. VS-U and VS-L were all significantly different among three VD angles, and decreased as the VD angle increases. In present study, as the VD angle increasing, VS-U was different from VS-L, and VS-U was higher than VS-L, which showed that vertebral body was more prone to fracture when the load was perpendicular to the lower edge of the vertebral body as the VD angle increasing. This may be due to the fact that the angle between the upper edge of L1 and the Y-axis line is usually greater than the angle between the lower edge of L1 and the Y-axis line after the vertebral wedge deformity (as shown in Fig. 5, angle α usually greater than angle β). Why is angle α usually greater than angle β, the reason may be that, from the perspective of biomechanical analysis, the force on the upper edge of the vertebral body should be consistent with the lower edge, however, when the force on the lower edge may be buffered when it comes into contact with structures like the disc reducing the reaction force, so the deformation degree of the upper edge was slightly heavier. Thus, as shown in Fig. 5, the load a is equal to the b, the force leading to the anterior and posterior displacement of the vertebral body in the Y-axis direction is small and can be ignored, and the Z-axis component force of load b is greater than load a because of angle α greater than angle β. Thus, when load b is applied, the displacement of L1 is larger and the VS is smaller. Or this may be pseudomorph caused by the removal of vertebral body accessory, intervertebral disc, ligament muscle and other structures.

Several limitations should be discussed. Firstly, the vertebral body accessory, intervertebral disc, ligament muscle and other structures were removed, and only the vertebral body was analyzed for QCT-FEA models. Secondly, only three VD angles of QCT-FEA models were reconstructed. While now, FEA of each patient’s clinical-resolution computed tomography scan, accounting for variations in geometry, cortical thickness, and material properties to assess bone stiffness and bone strength [35], is now available in the USA as a Medicare screening benefit for osteoporosis diagnostic testing, helping to address under-diagnosis of osteoporosis [21]. However, the process is complicated and time-consuming. We focused on the influence of VD on VS, so simplified the model and only three different VD angles were analyzed. Thirdly, only loads in two directions and only VS were analyzed in QCT-FEA, experimental results had not been verified in vivo, and only VS was analyzed without vertebral strength or follow-up of fracture probability. Experimental stiffness of the whole vertebral body was measured as the slope of the linear portion of the load–displacement curve, i.e., between 26 and 56% of the peak force, and experimental strength of the vertebral body was defined as the peak force at the load–displacement curve [22]. Through previous studies, the vertebral stiffness has a formula consistent with the results of the carcass analysis, and can also reflect the state of vertebral body [22]. Finally, this was a single-center study and the sample size of 70–80 years is slightly smaller.

## Conclusions

We demonstrated that VD angle of L1 was slightly increased with age and not correlated with vBMD, and VS was moderate negatively correlated with age, showing that the vertebral body was more likely to fracture with aging. VS-U and VS-L were gradually decreased with the increase of VD angle, and VS-L was lower than VS-U with the increase of VD angle, which showed that vertebral body was more prone to fracture when the load was perpendicular to the lower edge of the vertebral body as the VD angle increasing.

## Supplementary information

**Additional file 1.**

## Data Availability

All data generated or analysed during this study are included in this published article and its supplementary information files.

## Abbreviations

VD: Vertebral deformity
VS: Vertebral stiffness
QCT: Quantitative computed tomography
vBMD: Volume bone mineral density
BMD: Bone mineral density
QCT-FEA: QCT-based finite element analysis
VD angle: Vertebral deformity angle
L1: 1^St^ lumbar vertebral body
BMI: Body mass index
ROI: Regions of interest
HU: Hounsfield unit
VS-U: Vertebral stiffness when the load perpendicular to the upper edge of 1^st^ lumbar vertebral body
VS-L: Vertebral stiffness when the load perpendicular to the lower edge of 1^st^ lumbar vertebral body
